# Supplementary material for: A Rapid and Accurate MinION-Based Workflow for Tracking Species Biodiversity in the Field
Source: Genes (Basel). 2019 Jun 20;10(6):468. doi: 10.3390/genes10060468 (PMC6627956; doi:10.3390/genes10060468)
Supplement: Supplementary file 1 [file genes-10-00468-s001.zip › Supplementary_Files/Figure S1.docx]

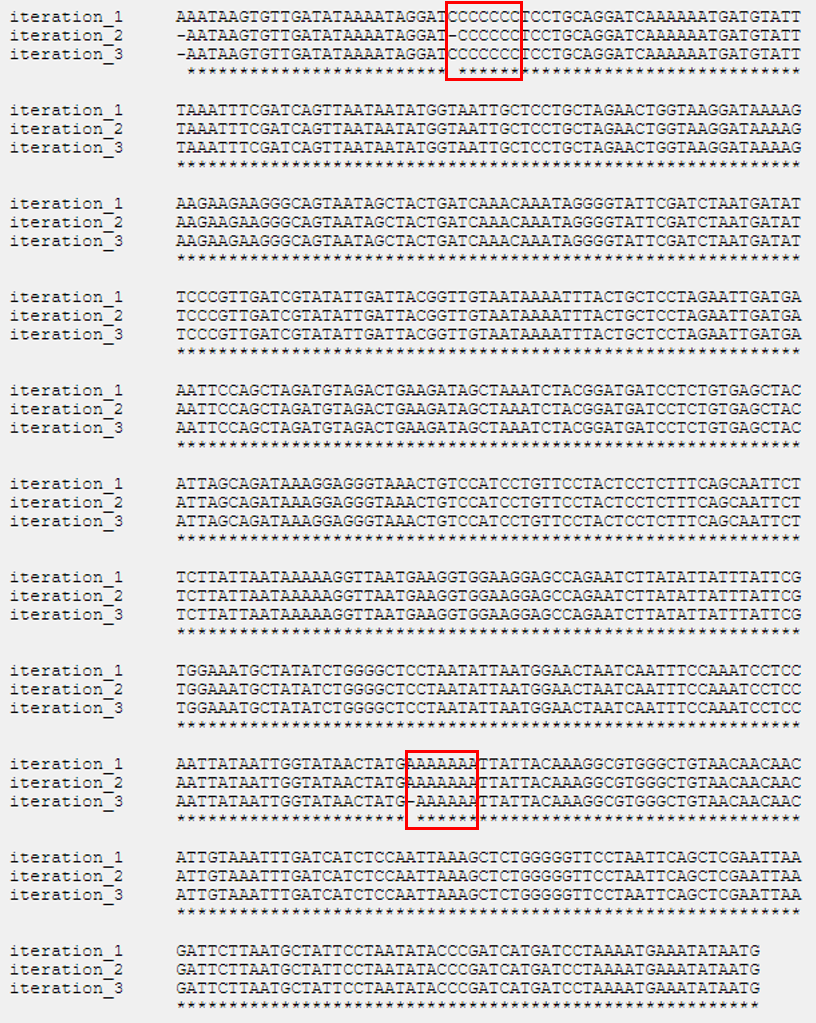


**Supplementary Figure S1. Analysis of differences between the three consensus sequences generated for sample BC03.** Multiple alignment of the consensus sequences generated from three iterations for sample BC03 is shown. The differences between the three sequences, present in homopolymeric runs of 7 nt, are highlighted in red.
